# Book Reviews

**Published:** 1800-05

**Authors:** 


### CRITICAL RETROSPECT

### OF

### MEDICAL AND PHYSICAL LITERATURE.

### [ FOREIGN AND DOMESTIC. ]

---

*An Inquiry into the Symptoms and Causes of the Syncope Anginosa, &c.* *By* C. H. PARRY, M. D.

[ Concluded from our last Number, pp. 389—390. ]

" As the disease advances, or in violent cases, the paroxysms sometimes come on, or are much increased, from certain passions of the mind; from slow walking; from riding on horseback, or in a carriage; from swallowing, speaking, coughing, or straining at stool; and sometimes also they attack the patient from about two to four o'clock in the morning, or while sitting or standing, without any previous exertion or obvious cause. The paroxysms now also become more violent, and do not so readily recede. During the fit, the pulse sinks in a greater degree; the face and extremities become pale, and bathed in a cold sweat, and for a while, perhaps, the patient is deprived of the powers of sense and voluntary motion. At length, after the disease has recurred more or less frequently, sometimes during a space of many years, which admit of the patient's death from a variety of other causes, a more violent attack, of the nature which I have just described, puts a sudden period to his existence.

" These are the essential symptoms and more obvious causes of the unmixed Angina Pectoris.

" To this we may add, that the Angina Pectoris is in no stage attended with inflammatory fever, and that both its termination, and the appearances, on dissection, of those who die of it, are totally different from those related in the paper (to which I refer) in the London Medical Transactions.

" Equally dissimilar also to the disease which I have described are those three cases by Drs. Macbride and Smith, of Dublin, in the fifth volume of the Edinburgh Medical Commentaries. They are evidently cases of palpitation of the heart, such as every physician of extensive practice must have often seen. In almost every violent case of this kind, there is a pain of the chest and elbows, as in the true Angina Pectoris. Nor is it difficult to understand how a rapid and irregular transmission of blood through the carotid and pulmonary arteries should produce that laborious respiration, turgescence and redness of the face and eyes, and head-ach, which which are mentioned in the cafes referred to. In the true Angina Pectoris, on the contrary, as we have feen above, there is neither dyfpnœa nor palpitation of the heart.

"From the detail which I have given, it appears that there have been publifhed not more than ten effays relative to the true Angina Pectoris, containing only as many detailed cafes, and nine diffections of perfons dying of that diforder.

"We cannot wonder that an experience fo contracted fhould have left fome fymptoms of the difeafe unnoticed, and much uncertainty with regard to the diftinctions and pathology. Thefe deficiencies, I truft, will be in part fupplied by the cafes which I have related.

"In Mr. Bellamy, the Angina Pectoris appears to have been much complicated with another diforder, from which its fymptoms are fcarcely feparable; but the two laft cafes are by far the moft fimple of any which have been detailed. They had medical affiftance the fooneft after the commencement of the paroxyfm, and proved fatal after the fmalleft number of attacks. Mr. S**** may be almoft faid to have twice died; fo that the circumftances accompanying the fatal termination were capable of being afcertained in the moft exact manner.

"He had the common fymptom of a pain affecting the fternum, and extending from thence acrofs the lower part of the left mamma, firft into the infide of the left elbow, and afterwards of the right elbow. This pain was relieved by eructations. He had no dyfpnœa, or palpitation of the heart. His pulfe was weak and fmall; and had, at long intervals, an occafional imperfect ftroke. Thefe fymptoms have already been mentioned by authors as generally occurring. The following circumftances I cannot any where find defcribed:

"My patient's diforder was increafed by bending the trunk of the body forwards; and it was probably from fome relief which he experienced that he was fond not only of ftraitening the fpine, with the head fomewhat reclined backwards, but alfo of ftretching out his arms in the pofture of yawning. He fighed frequently, and feemed to take great pleafure in refting on a full infpiration, which afforded a momentary relief to the uneafy fenfation in his breaft. Is it poffible that this fymptom, which is not remarked by any of the writers on the Angina Pectoris, was wanting in thofe cafes which fell under their notice? I am difpofed to think that it was not; becaufe it has been very obfervable in feveral examples which I have known of patients labouring under this difeafe; and my learned friend, Dr. Falconer, with whom I have converfed on this fubject, affures me, that it was particularly remarkable in two inftances which were fome years ago under his care, nearly at the fame time, and which ended in fudden death.

"From the preceding obfervations, I think it evidently appears, that the Angina Pectoris is a mere cafe of Syncope or Fainting, differing from the common Syncope only in being preceded by an unufual degree of anxiety or pain in the region of the heart; and in being readily excited, during a ftate of apparent health, by any general exertion of the mufcles, more efpecially that of walking.

"On this principle I would thus venture to infert this difeafe, in Dr. Cullen's Nofological Syftem, under the trivial name of Syncope Anginofa.

"G. XLIV. SYNCOPE.

"Motus cordis imminutus, vel aliquamdiu quiefcens.

"I. IDIOPATHICÆ.

"1. Syncope (*cardiaca.*) *Ex. vitio cordis, vel vaforum vicinorum.*

"a. *Anginofa.* A corporis motu inter ambulandum fæpe oriens; præeunte anguftia, vel dolore, pectoris notabili, per mammam finiftram præcipue porrecto; fine cordis palpitatione.

"Angina Pectoris Auctorum.

"b. *Palpitans.* Sine caufâ manifeftâ fæpe rediens, cum palpitatione cordis vehementi in intervallis.

"2. Syncope (*occafionalis*) ex affectione totius fyftematis manifeftâ.

"II. SYMPTOMATICÆ, five fymptomata morborum, vel totius fyftematis, vel aliarum præter cor partium.

"Befide fome other changes in Dr. Cullen's original claffification, I have, in this new arrangement, inferted the Syncope Anginofa as a variety of the Syncope Cardiaca; and I have given the trivial name *palpitans* to that arifing from the more common difeafes of the heart, becaufe the latter is ufually attended with palpitation, which I have marked as wanting in the former."

Chap. IV. p. 70, is entitled, "Caufes of Syncope in general.—Diffections.—Predifpofing Caufes.—Exciting Caufes.—Caufes of *Syncope Anginofa.*—Difeafed Coronary Arteries of the Heart."

But it is unneceffary for us to give any farther extracts from this work, as we are convinced that every medical practitioner will fpeedily and carefully perufe the original.

---

*The Anatomy of the Gravid Uterus, with practical Inferences relative to Pregnancy and Labour. By* JOHN BURNS, Surgeon, Glafgow, 8vo. pp. 248. price 5s. in boards. London, Johnfon.

We have no hefitation in recommending this work to our readers. In the Introduction the author juftly infifts on the neceffity of an accurate anatomical knowledge of the gravid uterus, as the foundation of the art of midwifery; and truly obferves, that, "He who his ignorant of this fubject, can only practife with impunity in thofe cafes where the affiftance of art is ufelefs; and even here, he is only fafe whilft he remains a mere fpectator." But the author does not confine his obfervations folely to the anatomy of the uterus; the reader will find confiderable attention has been paid to the pathology of this organ, and alfo feveral important practical directions are given on the diforders attendant on pregnancy, as well as on actual labour and its confequences.

On the fubject of flooding occurring about the feventh month, and which takes place from the attachment of the placenta over the the os uteri, we ſhall give the author's own words, as they are an epitome of the beſt modern practice.

"When flooding depends upon this cauſe, veneſection, cold, and the uſual remedies, may moderate or check it for a time; but the only radical cure is delivery. This, however, is at firſt difficult, or impoſſible to be accompliſhed, from the tightneſs of the vagina, and the firmneſs of the os uteri. The beſt practice, therefore, is, to reſtrain the hæmorrhage, by cold applications, or a plug, until the parts will more readily admit of diſtenſion. Until this can be done, the danger is not great, becauſe, as long as the os uteri is firm and ſmall, the bleeding is, comparatively ſpeaking, inconſiderable. In this ſpecies of flooding, the quantity of blood which is loſt marks the progreſs of labour, or the degree of dilatation; and whenever the flow is ſo great as to demand our immediate interference, we may be certain that delivery can readily be accompliſhed. The danger of the caſe, from immediate loſs of blood, and the eaſe with which we can operate, are exactly proportioned to each other. The propriety, therefore, of not interfering manually too ſoon, will readily appear; becauſe, at firſt, we may, by cold and plugs, moderate the hæmorrhage, until the parts admit of delivery; whilſt we ſhould inevitably increaſe the diſcharge, by beginning our operation prematurely, at the ſame time that we did not, by this conduct, gain one ſingle advantage.

"We then introduce the fingers, to dilate the os uteri, and either ſeparate the placenta, or puſh our hand through its ſubſtance. Puſhing the hand through the placenta is by no means ſo adviſeable as ſeparating it, where this can be done; becauſe the placenta, when attached over the os uteri, is generally leſs in circumference, and greater in thickneſs, than when attached at the fundus. We have, therefore, a great number of cells or veſſels to tear, and find it difficult to pull the child through the mangled placenta, which will continually interrupt us in our operation.

"After which, we lay hold of the feet, and deliver ſlowly. I ſay ſlowly, becauſe precipitation is uſeleſs, as well as dangerous, the body of the child acting as a plug, and reſtraining the bleeding.

"Delivery then, is the only chance of ſafety, and this we begin as ſoon as the ſtate of the parts will permit us. Evacuation of the waters, which is uſeful in other ſpecies of flooding, is uſeleſs here, and ought never to be procured, unleſs as preparatory to delivery, when we are ready to perform it. The neceſſary prelude to this evacuation, namely, the ſeparation or piercing of the placenta, muſt increaſe the diſcharge, inſtead of abating it."

We would recommend to ſome of our Correſpondents, who have lately favoured us with remarks on Adheſion of the Placenta, to read what Mr. Burns has written on this ſubject.

*The most Cogent Reasons why Astringent Injections, Caustic Bougies, and Violent Salivations, should be banished for ever from Practice: with the mildest Methods of safely treating every Species of Venereal Infection, Strictures of the Urethra, &c. and correcting Mischiefs arising from Caustic Bougies.* By William Rowley, M.D. Member of the University of Oxford, &c. Large 8vo. pp. 180. Price 4s. London. Murray and Highley.

The author's celebrity, and the importance of the subjects treated of in this work, will not fail to procure it readers. We cannot present a better account of the contents of this performance, than that given by the author in the Conclusion.

"THE origin, progress, improvements, and treatment of every species of venereal infection have been discussed, as far as this performance admitted. Animadversions on destructive principles and practice, new or old revived chimerical projects, have been profusely introduced. Antivenereal remedies, whether openly avowed and regular, or secret and irregular, have been examined and criticised. The reasons for never treating any venereal infection without mercury, and the necessity of banishing powerful salivations, are submitted to the consideration of the learned and experienced in the medical profession: they are founded in reiterated observation, and admitted by the most able practitioners in Europe. The merits of mild successful practices, and the additional force of mineral alteratives in complex venereal cases, according to circumstances, constitutions, seasons, and climates, are energetically inculcated. Many original and appropriate prescriptions have been communicated, for the different purposes of practice in the variety of circumstances that occur. The superior utility of fumigations, and of many methods of treatment, has been defended, which fleeting whimsical hypothesis, or which inexperience or presumption, had rashly decried, without any practical knowledge of their importance. Astringent injections have been demonstrably proved the principal cause of urethral obstructions and stricture. Mild methods of treating such cases, with safety and success, have been ardently recommended and fully authenticated. Caustic bougies, applied to the urethra under pretence of removing strictures, have been shown not only inadequate to their intentions, but often dreadfully destructive in their consequences. When radical cures have been confidently promised by the causticators, and credited with the warmest hopes and expectations by the deluded patients, additional misery, permanent sufferings, or a dreadful death, have been too often the consequences of the tormenting delusion. The methods of alleviating the horrid miseries, which caustics produce when applied to the exquisitely sensible urethra, are lastly introduced, and many causes of impotence from urethral complaints explained. The whole work is interspersed with sentiments and reflections on many defects of the art, and what appeared the most effectual means of their removal, by a learned and practical education, such as Boerhaave, Hoffman, Heister, and other distinguished physicians and surgeons have possessed and inculcated. These doctrines doctrines are chiefly intended for the ſerious peruſal and contemplation of ſtudents; and the inexperienced, and all who have been deluded by thoſe and other late chimerical fallacies, which have urged the neceſſity of the preſent animadverſions.

"Having now diſcharged a duty to the profeſſion, and to the public, from whom, through a long life, ſo much confidence and protection have been experienced, a ſhort pauſe may be requiſite.—An apology may be thought expedient.—An apology ſhould be offered for the free cenſures advanced; the frequent repetitions of which, to ſome readers, may prove diſguſtful. To the learned and well-informed, many parts may be thought redundant; but to thoſe who are inexperienced, and have yet their profeſſion to learn, a work cannot be too explicit or inſtructive. There appeared but two modes of procedure on the preſent occaſion; either ſilently to ſuffer a continuance of dreadful injuries to ſociety, or to openly expoſe their evil tendency. The former would have been puſillanimous and iniquitous; the latter, therefore, has been adopted, in order to reform ſome of thoſe ſerious abuſes, that have been revived or forced into the art with more ſpecioufneſs and raſhneſs than truth.

"The cenſures are not levelled againſt any particular practitioners; but formed on the broad baſis of general and public utility. Medical men ſhould live in the utmoſt harmony and eſteem, and never feel offended for difference of opinion. Facts alone ſhould decide, and ſagely direct their practical conduct. An affection, a warm affection for the excellent art of ſurgery, and a ſincere regard for its honour, prompted the abſolute neceſſity of the preſent publication. Numerous practitioners, both in town and country, are well informed of the deleterious effects announced, and ſome may be found, more capable of treating theſe important ſubjects. Many, who vehemently condemn in private converſation the practices impeached, ſhrink from the ungrateful taſk of publicly expoſing the direful conſequences. Several have expreſſed an impatient deſire to ſee the recited errors brought forth to public view, and, if poſſible, baniſhed; yet they ſhift the ONUS SCRIBENDI on any ſhoulders except their own. It is more laudable to attempt to ſerve the public with moderate talents, than to ſuffer horrid miſery to be inflicted on mankind, under the ſeducing appearance of the greateſt benefits.

"It would argue great apathy and negligence, after immenſe experience, joined to an ardent attachment to the art for above forty years, not to preſent all the fruits of conſtant induſtry and attentive inquiry. It will appear, on reflection, that I come not to deſtroy, but to ſave.

"Thoſe, who have been attached to the uſe of aſtringent injections, have been proved to be the ringleaders of the moſt terrible evils, either immediate or remote, that ever afflicted mankind. Relinquiſh, then, that favourite practice, that injurious prejudice: thus will urethral ſtrictures daily be reduced in number, and, in time, be heard of no more!

"As

" As the evils of urethral ſtrictures muſt occur ſo long as the effects of aſtringent injections, or other mal-practices continue; let not the cruel violent modes of burning the moſt ſuſceptible parts of man be adopted for their removal.*

" It may be expected that this work, however dictated by humanity, muſt produce private enemies, who will ſecretly whiſper what they dare not openly avow. A man accuſtomed to the detraction of malevolence for public benefits has little to apprehend; but mankind ſhould be warned how they receive calumniating inſinuations inſtead of truth. In this liberal ſcience let not the proverb be applied, FIGULUS FIGULUM ODIT, MEDICUS MEDICUM. However events may happen, it can only be ſaid, that, unleſs provoked, many additional proofs of the miſchievous tendency of the cenſured doctrines ſhall ever be ſuppreſſed; but if forced, by any defence of the practice, they ſhall appear in all their hideous colours."

---

*Annals of Medicine for the Year* 1799: *exhibiting a conciſe View of the lateſt and moſt important Diſcoveries in Medicine and Medical Philoſophy.* By ANDREW DUNCAN, Sen. M. D. and ANDREW DUNCAN, Jun. M. D. Fellows of the Royal College of Phyſicians, Edinburgh. Vol. IV. 8vo. pp. 580. Price 8s. in boards. London, Robinſons; Edinburgh, Mudie.

THIS volume is divided into four Sections. The firſt, which occupies about half the volume, contains an analyſis of books; and of thoſe publiſhed in England we have already given an account in our preceding numbers; of the foreign articles, an analyſis will be given as ſoon as poſſible.

The ſecond ſection is intitled *Medical Obſervations,* compriſing original communications of caſes, and their treatment; ſome of which are referred to in the preſent † and preceding ‡ numbers of this Journal.

The ſecond, in the order they are publiſhed, is a *Caſe of Uterine Hæmorrhage, where the Placenta was expelled four hours before the Birth of the Child.* By Mr. JOHN CHAPMAN, Surgeon, at Ampthill. As there is ſomething uncommon in this caſe, we ſhall preſent our readers with an abſtract of it.

" Upon my ſecond examination," ſays the author, " I diſcovered the head, as before, with an edge of the placenta beginning to protrude

---

* A diſtinguiſhed nobleman was ſaid to be perfectly cured by the burning cauſtic—pus, ſanies, and different coloured matter is daily voided with the urine, with additional calloſities in moſt parts of the urethra.

Such are the pretended cures, many inſtances of which I have ſeen. A ſervant of the ſame nobleman loſt his life by the cauſtic.—Whilſt I am writing this, a clergyman is preſent, who unhappily had the cauſtic repeatedly applied, in the courſe of two or three years; the original cauſe of his ſtricture was the application of aſtringent injections, when a youth at Eton. The uſe of cauſtic has rendered him truly miſerable, who was but ſlightly ſo before.

A gentleman had a cauſtic lately applied to the urethra; cold ſhiverings followed, and he died in a few hours.

† See Mr. Hull's Caſe, page 428. ‡ See Vol. III. page 322.

trude through the os uteri, with a very trifling hæmorrhage. This was increafed upon the return of the pains, but was fo inconfiderable, as not to be directly alarming. I therefore did not conceive myfelf juftified at this ftage in proceeding to immediate delivery; but as, upon every return of pain, the placenta became more and more protruded through the os uteri; and as it was now entirely detached, without the head in the leaft advancing, and the hæmorrhage being fomewhat more increafed, I informed her of her fituation, and the neceffity there might be for immediate delivery, if the hæmorrhage increafed: Finding it would be with very great reluctance fhe would agree to this, I requefted my worthy friend, the late Mr. Humberftone, (the gentleman I then affifted), might be fent for. Previous to his arrival, the pains continued fo ftrong, that the os uteri became dilated, and the placenta was completely expelled through the os externum: this happened about three o'clock on Monday morning, and with very little hæmorrhage; but from this moment the pains entirely ceafed. I would now have proceeded to delivery, but it was objected to, becaufe fhe had not any pains; that fhe had plenty of ftrength; and that they hoped there would not be any impropriety in waiting the arrival of Mr. H., who was every moment expected: I agreed, if no hæmorrhage happened in the mean time. He did not arrive until five o'clock. There had not been the leaft hæmorrhage fince the expulfion of the placenta. We now concluded, as the pains had entirely fubfided, much was to be feared, and nothing could be gained, by delaying delivery any longer. If we were to wait the return of pains, the hæmorrhage might return, and we fhould be brought to that point of time when we muft be obliged to deliver; and fhe would moft probably die under the operation. Having great ftrength and fpirits, the neceffity of turning was properly reprefented to her; and the danger that might accrue from any farther delay. She now fubmitted, and the parts being perfectly well dilated, Mr. H. introduced his hand, with little difficulty, into the uterus; but with all his efforts, for upwards of half an hour, he could not recover a foot. I then made an attempt, and introduced my hand readily into the uterus, and found it fpafmodically contracted, in a longitudinal direction; the circular fibres acting without the confent of the longitudinal. I proceeded with the greateft degree of caution, acting betwixt the pains, (which our efforts had now excited); but every effort to introduce my hand farther towards the fundus uteri, where the feet evidently lay, increafed the fpafmodic contraction, that with all my efforts for nearly an hour, I gained nothing, and defifted. During the whole of this time, the hæmorrhage had not in the leaft increafed. Our efforts to turn being thus fruftrated, we thought that, by bringing down the hand, the head might be brought fufficiently low in the pelvis, (being a large pelvis, and only an eight month's fœtus), as to come within the grafp of the forceps, refolving to apply them higher than would be admiffible in almoft any other cafe. The forceps were then fent for; but in the interim, it was propofed by Mr. H. to give a dofe of tinct. opii, to take off this this preternatural action of the uterus. I objected to it, as we could not say, Go so far, and no farther; and if it should so far take off the action of the uterus, and the hæmorrhage return, we should most probably lose our patient. However, I consented to twelve drops *only* being given. In a very short time, she became easy and comfortable. In less than half an hour, the natural pains returned; so that six or seven pains expelled the child entirely by the efforts of the mother, the head and arm presenting. Nothing remarkable happened in her recovery, except in about ten days, she suffered triflingly from the swelled leg, (which has been so well described by Mr. White). I must deviate from my subject, to observe, that it is the opinion of some very respectable men, that this never affects the same patient more than once; but this woman had the same affection of the leg in her first labour.

" REMARKS.

" 1st, What I wish to call the attention to in the above case is, that, notwithstanding the placenta was nearly three hours from the first protusion through the os uteri to its complete expulsion through the os externum, she lost very little more blood than women usually do when the placenta is expelled after the birth of the child.

" 2dly, From the expulsion of the placenta to the birth of the child, was full four fours. She lost *little* or no blood. How far does this suggest a different practice, (to that in general followed), I mean, of delivering the placenta previous to delivering the child, in those cases of alarming hæmorrhage, where the placenta is situated on the side of, or over the os uteri?

" 3dly, The very singular and sudden effect produced by so small a dose of tinct. opii, in removing the spasmodic contraction of the uterus, suggests to us a most excellent remedy on these occasions."

---

The following extract from page 492, &c. is given in order to illustrate the communications of Drs. Trotter and Yeats, on Nitrous Fumigation, in this and the preceding number. In a letter to Dr. Duncan, Dr. Yeats says:

" In turning over the pages of the last volume of Annals of Medicine, I find another testimony in favour of Dr. C. Smyth's plan for destroying contagion. As a further proof of this, I can add, that it was successful in arresting the progress of a contagious fever in the jail of this place. Three persons were taken ill of a typhous fever, one of whom died. This circumstance reached the ears of a gentleman of great respectability in this neighbourhood; he was immediately prompted by his well-known humanity, to order further assistance, and desired me to attend. Only one person was ill of the fever when I went, but he was dangerously so, and the contagion was evidently attacking another. All the rooms were immediately fumigated with the nitrous vapour. The man ill of the fever shewed symptoms of amendment very shortly; and in the other, other, on whom the typhous contagion had commenced its attack, no further marks of the complaint made their appearance. I should have laid no stress upon these circumstances, had I not been informed, that many years ago, a contagious fever appeared in the jail here, and spread into the town, of which several died. It appears to me, from what I observed, that had not the nitrous fumigations nipt the contagion in the bud, something similar might have happened at the present time. I beg leave to subjoin a few remarks on the objections which Dr. Trotter has made to Dr. Smyth's plan. In the Medicina Nautica, p. 229, Dr. Trotter has observed, that 'Dr. Smyth's Preventive is the very substance that every intelligent officer is hourly employed to drive from the decks of his Majesty's ships.' Again, p. 230, 'In Dr. Smyth's process, when the nitrous acid is converted into gas, it loses a portion of pure air; it is now an elastic fluid, under the title of nitrous air, or gas. In this state it will remain for some time, till it again, by a chemical attraction, recover its pure air, when, by its specific gravity, it will fall to the deck, nitrous acid.' Were this statement correct, the inference no one could deny; but it is not *nitrous gas* which is disengaged, but concentrated *nitric acid* in a *state of vapour*. In this state it has a large quantity of oxygen in its composition. Instead, therefore, of abstracting that principle, it will readily part with it, and thus render the atmosphere purer. The fumes procured from nitre by the sulphuric acid, are very different in their appearance and qualities from nitrous gas. The former is a white visible vapour, but nitrous gas is invisible; and when set free, immediately unites with the oxygen of the atmosphere, and forms the orange-coloured nitrous vapour. There is, therefore, a material difference between the two This mistake, Sir, seems to have escaped the notice of all who have engaged in this controversy between Dr. Smyth and Dr. Trotter. There is no doubt, that the *nitrous gas* would be extremely prejudicial, but the *nitric vapour* is certainly useful, and possesses qualities diametrically opposite to the nitrous gas. Dr Trotter's objections to Dr. Smyth's plan, appear to have arisen from his not having a perfectly distinct chemical idea of the nature of these gases. Dr. Trotter observes, page 229, 'That in proportion to the quantity azot attracts of oxygen, it is called azot, azotic gas, nitrous gas, nitrous acid, nitric acid.' Again, p. 239, 'There is no great difference between azotic and nitrous gas.' The mistake here is evident; azot and azotic gas contain no oxygen. This principle is termed simply azot, when freed from its caloric; but when it assumes the aeriform state by a combination with the matter of heat, it is called azotic gas. With respect to the difference between azotic and nitrous gas, there is a very material one. The azotic gas contains no oxygen, and does not readily unite with it, as every one knows who has attempted to form the nitrous acid, by passing the electric spark through a mixture of azotic and oxygen gases. Nitrous gas, on the contrary, is a compound containing oxygen, and readily uniting with it to form nitrous acid at the temperature of the atmosphere. A candid

 examination examination will convince a mind so open to philosophical conviction as Dr. Trotter's, that a misconception of this kind has led him into errors in his objections to Dr. Smyth's plan. The health of his Majesty's naval subjects is intimately connected with the dispute; and I trust, that nothing but a zeal for the improvement of science will induce any person to attach himself to the one or the other side."

---

*A Letter to Thomas Keate, Esq. Surgeon General to the Army, one of the Surgeons to St. George's Hospital, &c. &c. with some Remarks on the Medical Profession, occasioned by the approaching Election of a Surgeon to St. George's Hospital, vacant by the Resignation of Charles Hawkins, Esq. on the 9th of April,* 1800, pp. 32. Hurst, 1s.

The purport of this letter is to prove that the Governors at large are not competent judges of the merits of the candidates. The author wishes some alteration to be adopted, and " is sure the business is best conducted by committees, which should be elected annually, at a general meeting of the governors."

That the committee of twenty-four professional and extra professional members, should consider the merits of the candidates for any vacant offices, and for those of surgeon and physician, and be empowered to elect successors, who, the author proposes, should have more than two-thirds of the whole committee in their favour.

